# Regulatory effects of miRNA on gastric cancer cells

**DOI:** 10.3892/ol.2014.2232

**Published:** 2014-06-05

**Authors:** OUYANG YANG, JIANHUA HUANG, SUN LIN

**Affiliations:** Department of General Surgery, Xiangya Hospital of Central-South University, Changsha, Hunan 410008, P.R. China

**Keywords:** gastric cancer cell, gene regulation, microRNA

## Abstract

The present study aimed to screen the regulatory mechanism of microRNA (miR/miRNA) typing on gastric cancer cells in gastric cancer tissues. In total, 46 patients who underwent gastric resection in Xiangya Hospital of Central-South University (Changsha, Hunan, China) between January and December 2012 were selected. Gastric cancer cells were obtained for RNA extraction, and miRNAs were detected by quantitative polymerase chain reaction. Compared with the expression levels in the normal gastric mucosa, the expression levels of miR-9, miR-433, miR-19b and miR-370 were downregulated; the differences was statistically significant, except for miR-19b (P<0.05). miRNAs play a regulative role in the occurrence and development of gastric cancer, and changes in their expression provide a basis for the diagnosis of this disease.

## Introduction

Gastric cancer is one of the most common malignant tumors in the digestive tract. In China, almost 300,000 individuals succumb to gastric cancer and ~400,000 new cases are diagnosed each year (1). The diagnosis, metastasis and prognosis of tumors use various molecular markers, so the search for molecular markers at different stages of tumor development is of great clinical significance to the early diagnosis, recurrence, metastasis prediction and prognosis of tumors (2). Studies on tumor markers have been carried out for numerous years, and there are also a small number of markers used in the clinic, but their sensitivity and specificity remain extremely limited (3,4). Therefore, it is essential to study the molecular markers associated with gastric cancer metastasis and prognosis. The emergence of microRNA (miR/miRNA) provides a novel concept for cancer research and the screening of molecular markers.

miRNAs are a class of endogenous non-coding small molecule RNAs with regulatory function on genes, which are 22–28 nucleotides in length and widely exist in eukaryotes (5). A gene with miRNA encoded in the nucleus is first transcribed into a pri-miRNA, i.e., the precursor of miRNA (6). Specific miRNAs have a close association with tumors. It has been confirmed that there are ~500 miRNAs in the human genome, of which at least 200 miRNA sequences are closely associated with tumors, and a number of them can be used as tumor molecular markers (7). miRNAs as molecular markers have unique advantages; they are more stable compared with mRNA, are not affected by other physiological states of the body, can be amplified and can be more easily detected compared with proteins (8).

The present study aimed to conduct screening, identification and validation of miRNAs in gastric cancer tissues and cell lines, combined with an analysis on the clinicopathological factors and survival data of gastric cancer patients. The study also aimed to reveal the regulation mechanism of miRNA expression in gastric cancer, in order to provide a theoretical basis for miRNAs to become novel molecular markers and therapeutic targets of the metastasis and prognosis of gastric cancer.

## Materials and methods

### Materials

The following materials were used in the present study: A normal gastric mucosal epithelial cell line, GES-1 (Shanghai Cell Bank Center, Chinese Academy of Medical Sciences, Shanghai, China), a gel imager and electrophoresis apparatus (Bio-Rad, Hercules, CA, USA) and pipettes (5, 10 and 200 μl and 1 ml; Eppendorf, Hamburg, Germany).

### Subjects

A total of 46 patients who underwent gastric resection in Xiangya Hospital of Central-South University (Changsha, Hunan, China) between January and December 2012 were selected. In total, thee study group consisted of 26 males and 20 females aged between 30 and 70 years old, with the mean age of 52.1±6.7 years old. The clinical data of the patients with gastric cancer were recorded completely, including pathological type, stage, metastasis, one-, two- and three-year survival rates and other detailed information. The study was approved by the ethics comittee of Xiangya Hospital of Central-South University. Consent was obtained prior to examination for the enrolled patients according to the Declaration of Helsinki and relevant laws in China. All treatments were performed based on the best interests of the patients.

### RNA extraction

The gastric cancer cells were plated in 6-well culture plates 24 h prior to transfection. When the cells reached 30–50% confluency, 10 μl antisense oligonucleotide liposome (Promega Corporation, Madison, WI, USA) was taken correspondingly and diluted to 250 μl by Opti-minimal essential medium (Invitrogen China Limited, Beijing, China) for action at room temperature for 10 min. The two were then slowly mixed prior to leaving the mixture stationary for 30 min and finally added it to the cells, which were agitated and mixed well. Following 12 h of culture, the medium was replaced with RPMI-1640 containing 10% fetal bovine serum for 48 h. Subsequently, the medium was aspirated, the cells were washed with phosphate-buffered saline and TRIzol (Invitrogen Life Technologies, Carlsbad, CA, USA) was added. The samples were then stored at −70°C (9,10).

### Quantitative polymerase chain reaction (qPCR)

The detection of the miRNA target and internal control, U6, was conducted in a Rotor-Gene 3000 Real-time PCR machine (Corbett Research, Mortlake, Australia). The 25 μl reaction system included 2.5 μl dNTP, 2.5 μl 10× PCR buffer, 1.5 μl MgCl_2_ aqueous solution, 1 unit Taq enzyme, SYBR-Green I with a final concentration of 0.25X, 1 μl PCR specific primer, 1 μl reverse transcriptase product and RNA-free enzyme water. The reaction conditions were as follows: 95°C for 5 min, followed by 95°C for 10 sec and 60°C for 1 min for a total of 40 cycles. The miRNA expression level was detected with the threshold cycle number (Ct). The miRNA targets were relatively quantified by the ΔΔCt method. ΔCt refers to the Ct value of the miRNA target minus the Ct value of U6RNA, and ΔΔCt is the value of the ΔCt value of each gastric tissue sample minus the ΔCt value of the normal gastric tissue sample. The changing multiple was calculated using the 2^−ΔΔCt^ formula (11).

### Statistical analysis

All data were analyzed by SPSS, version 13.0 (SPSS, Inc., Chicago, IL, USA). The measurement data were expressed as the mean ± standard deviation and processed by paired t-test and compatibility group design analysis of variance. P<0.05 was considered to indicate a statistically significant difference.

## Results

### RNA extraction results

**Figure 1:**
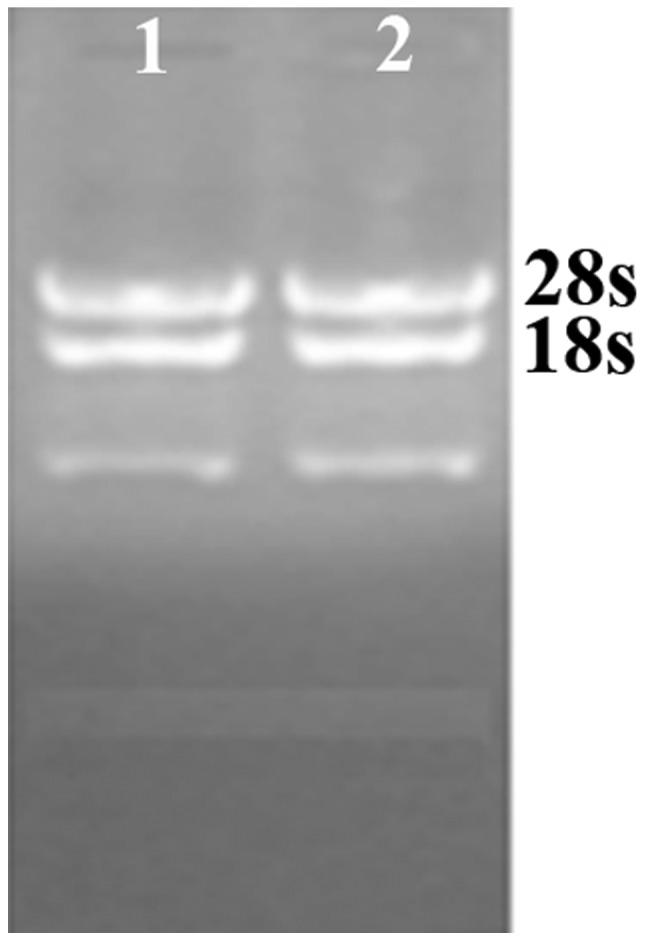
RNA extraction results.

The integrity of RNA can be assessed by electrophoresis on a denaturing agarose gel. Sharp 28S and 18S RNA bands (eukaryotic samples) are produced by intact total RNA that is run on a denaturing gel. An example for RNA electrophoresis on a denaturing agarose gel is shown in Fig 1. The RNA electrophoresis showed that the 28S and 18S bands were wider compared with the control (Fig. 1), indicating that the RNA was not degraded.

### Heat map and hierarchical clustering

**Figure 2:**
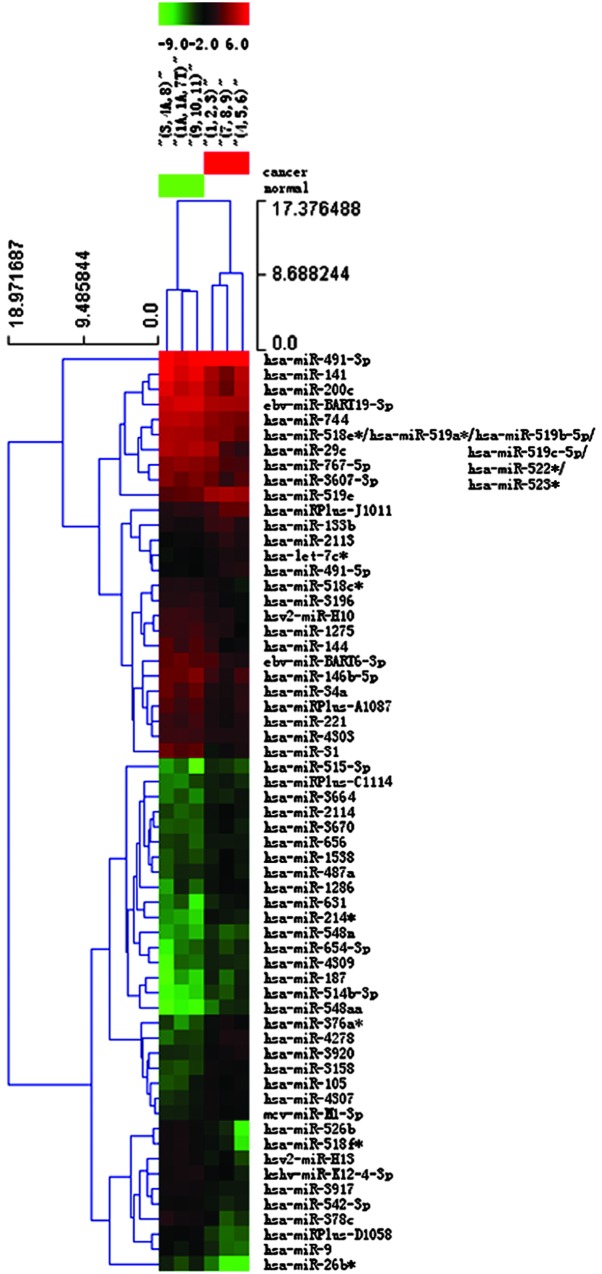
Hierarchical clustering for differentially-expressed miRNAs in a cancer vs. normal pass volcano plot (fold-change ≥2.0). Red indicates high relative expression and green indicates low relative expression. miRNA/miR, microRNA.

Two-way hierarchical clustering results for the miRNA and samples are shown in Fig. 2 by the heat map diagram. One miRNA is represented per row, and one sample per column. The left-hand side of the image shows the miRNA clustering tree, while the sample clustering tree appears at the top. Samples and miRNAs are arranged by cluster analysis into groups based on their expression levels, which enables hypotheses to be generated regarding the correlations between the miRNAs and samples. Subsequently, hierarchical clustering was performed based on the differentially-expressed miRNAs in a cancer vs. normal pass volcano plot. The result of hierarchical clustering shows distinguishable miRNA expression profiling among the samples (Fig. 2).

### miRNA expression in gastric cancer tissues

**Figure 3:**
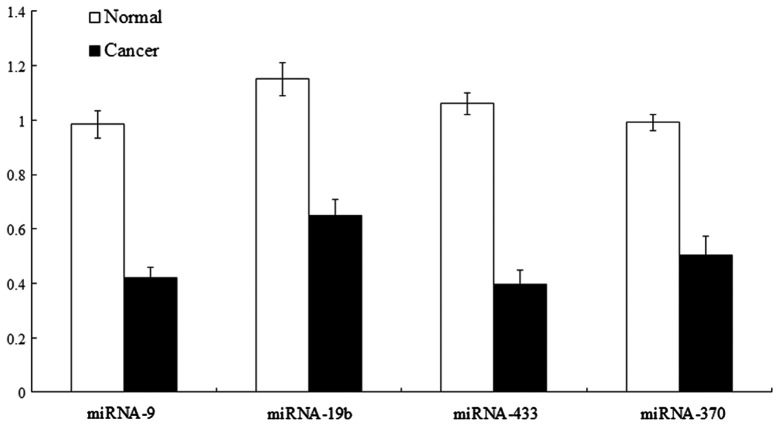
Expression levels of miRNAs in gastric cancer and normal gastric mucosa tissues. miRNA, microRNA.

**Figure 4:**
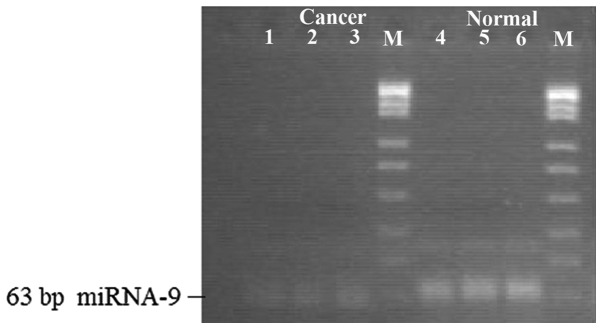
Expression levels of miR-9 in gastric cancer and normal gastric mucosa tissues. miR, microRNA.

**Figure 5:**
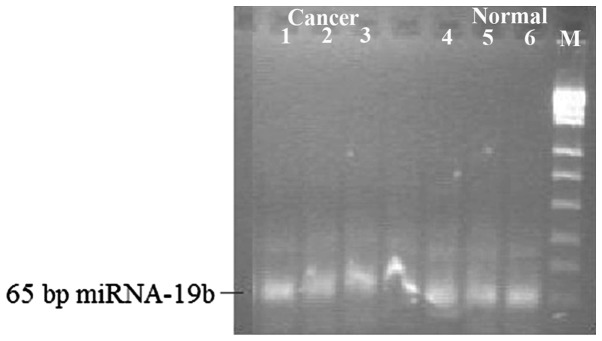
Expression levels of miR-19b in gastric cancer and normal gastric mucosa tissues. miR, microRNA.

**Figure 6:**
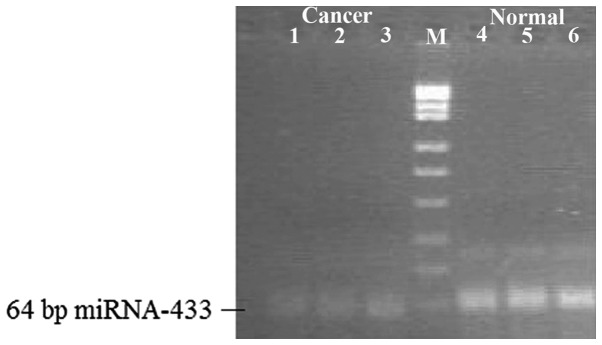
Expression levels of miR-433 in gastric cancer and normal gastric mucosa tissues. miR, microRNA.

**Figure 7:**
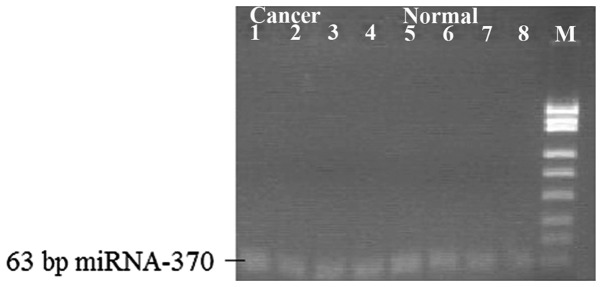
Expression levels of miR-370 in gastric cancer and normal gastric mucosa tissues. miR, microRNA.

The qPCR result showed that compared with the normal gastric mucosa, the expression levels of miR-9, miR-433, miR-19b and miR-370 were detected in the gastric cancer tissues and cells (Table I and Fig. 3), and the electrophoretic bands were located at 63, 64, 65 and 63 bp. Compared with levels in the normal gastric mucosa, the miR-9, miR-433, miR-19b and miR-370 expression levels were lowered (Figs. 4–7).

### Association between miRNA expression in gastric cancer and clinical pathological factors

Downregulated miR-9 expression was associated with the size of the foci and lymph node metastasis (P=0.036 and 0.028, respectively), while decreased miR-433 expression was associated with the foci site and pathological grading (P=0.003 and 0.005, respectively). Reduced miR-19b expression was correlated with the foci site and pathological grading (P=0.002 and 0.009, respectively), and downregulated miR-370 expression was associated with the foci site, pathological grading and lymph node metastasis (P=0.003, 0.004 and 0.004, respectively) (Table II).

## Discussion

There are various molecular markers that can be used for tumor diagnosis, metastasis and prognosis, therefore, the search for tumor molecular markers at these differing stages provides an important link between basic and clinical research, and a main way for basic research to serve a function in the clinic (12–14). Studies have shown that the metastasis and prognosis of gastric cancer is closely associated with the connexins (Cx). The mRNA and protein levels of Cx32 and Cx43 in gastric cancer cells and tissues are significantly reduced, and thus miRNAs that regulate Cx are molecular markers of gastric cancer (15,16). Through the process of looking for miRNA-9, -433, -19b and -370 which regulate Cx and via the use of statistical analysis on metastasis and prognosis in clinical groups, combined with clinical data, the present study confirmed that the miRNAs are good molecular markers. The present study is of great significance in the theoretical basis of the development, metastasis and prognosis of gastric cancer.

pri-miRNA is sheared into a length of ~70 nucleotides under the effect of the double-stranded RNA specific nuclease-Drosha RNase, then pri-miRNA, having a hairpin structure, is transferred from the nucleus into the cytoplasm with the action of the transporter protein, exportin 5, and cut into double-stranded miRNAs that are 21–25 nucleotides in length under the effect of another double-stranded RNA-specific nuclease, the Dicer enzyme (17–19). Subsequent to the unwinding of the double helix, one of the two chains connects with the RNA-induced silencing complex (RISC), functioning as an miRNA, and the other chain is degraded immediately. The combination of miRNAs with target mRNA can mediate the degradation of RISC into target fragments or hinder the translation process, which depends on the degree of mismatch between miRNA and the target mRNA 3′ untranslated region. If it is a complete or almost complete match, the RNA interference pathway can be induced and mRNA will be degraded. As the majority of miRNAs are not completely complementary with target mRNAs, which only play a role in closing mRNA targets and inhibiting the translation process so as to regulate the expression of target genes, miRNA plays a vital role in a variety of biological processes, including cell proliferation, differentiation and apoptosis (20–22).

In addition, through the application of gene-chip technology and bio-informatics methods, certain miRNAs that are closely associated with gastric cancer have been found. The experimental study by Li *et al* (23) showed that in 92% of gastric cancer samples, miR-21 had a significantly high expression level, indicating that miR-21 may serve as an effective diagnostic marker of gastric cancer. He and Wang (24) identified that in gastric cancer cell lines, the expression of the tumor-associated protein, high-mobility group AT-hook 2 (HMGA2), was inhibited by miRNA let27. In gastric cancer cells with a low HMGA2 expression level, the expression level of miRNA let27a, let27b and let27c was significantly higher compared with gastric cancer cells with a high HMGA2 expression level, indicating that miRNAs may play a biological role to promote or inhibit tumor development through the interaction between tumor-associated proteins.

The main factor affecting the prognosis of gastric cancer is the invasion and metastasis of gastric cancer, therefore, it is of great significance to study particular miRNAs that are closely associated with gastric cancer invasion, metastasis and development, which can also provide clues for the screening of miRNA molecular markers. A study by Li *et al* (25) analyzed the effect of miR-10a on the migration and invasion of the gastric cancer BGC823 cell line, and found that the transfection of miR-10a had no significant effect on the proliferation and apoptosis of BGC823, but could clearly promote its migration and invasion. Transfection with mature human miR-210a could increase the miR-10a expression in BGC823 and significantly promote the migration and invasion of BGC823 (26,27). Chen *et al* (28) studied the association between miRNA and RPL23 (a lymph node metastasis-related gene) by creating an miRNA expression plasmid that could express miR-155. This effectively silenced the expression of the PRL23 target gene in the gastric cancer SGC7901 cell line and significantly inhibited the invasion and metastasis of the SGC7901 cells. Therefore, miRNAs are closely associated with tumor proliferation and apoptosis, and their correlation with tumor invasion and metastasis requires further research.

To understand the carcinogenic mechanism of miRNAs, systematic identification of the target taking effect *in vivo* is required. The bioinformatics data show that each miRNA can regulate hundreds of target genes. It is estimated that 30% of human genes are subject to regulation by miRNAs, which also indicates that miRNA may affect all the signaling pathways (29). A study by Cao *et al* (30) selected myocardial cells as the study object and found that the regulatory targets of miR-1 existed in the GJA1-encoded Cx43, and miR-1 was overexpressed in myocardial cells and inhibited the expression of GJA1. Cx43 expression is reduced accordingly in the myocardium, aggravating arrhythmias (31). In this study, the expression of miRNA in gastric cancer tissues and cells was evaluated by analyzing the clinical pathological factors and survival data of patients. The findings revealed the mechanism regarding the mechanism of action, and provide theoretical evidence for the use of miRNA as novel molecular markers and treatment targets for the metastasis and prognosis of gastric cancer.

## Figures and Tables

**Table I:** Expression levels of miRNAs in gastric cancer and normal gastric mucosa tissues.

Tissue	miR-9	miR-19b	miR-433	miR-370
Cancer	0.419±0.211^a^	0.648±0.351^a^	0.397±0.284^a^	0.504±0.351^a^
Normal	0.985±0.429	1.051±0.589	1.061±0.514	0.991±0.512

a P<0.05 vs. normal.

miR/miRNA, microRNA.

**Table II:** Association between miRNA expression of gastric cancer tissues and clinical pathological factors.

		miR-9			miR-19b			miR-433			miR-370		
					
Clinical pathological factor	No. of cases	Low expression	High expression	P-value	Low expression	High expression	P-value	Low expression	High expression	P-value	Low expression	High expression	P-value
Gender, n				0.068			0.086			0.083			0.093
Male	26	23	3		22	4		23	3		24	2	
Female	20	18	2		18	2		19	1		18	2	
Age, n				0.077			1.021			1.132		1.324	
<50 years	24	22	2		21	3		22	2		21	3	
≥50 years	22	19	3		19	3		20	2		21	1	
Focus size, n				0.036			0.057			0.071			0.068
<3 cm	17	14	3		14	3		15	2		15	2	
≥3 cm	29	27	2		26	3		27	2		27	2	
Focus site, n				0.059			0.009			0.005			0.004
Lesser gastric curvature	28	25	3		27	1		27	1		27	1	
Greater gastric curvature	18	16	2		13	5		15	3		15	3	
Pathological grading, n				0.072			0.009			0.005			0.004
High differentiation	19	17	2		14	5		16	3		15	4	
Low differentiation	27	24	3		26	1		26	1		27	0	
Lymphatic metastasis, n				0.028			0.003			0.061			0.004
Yes	27	27	0		27	0		25	2		27	0	
No	19	14	5		13	6		17	2		15	4	

miR/miRNA, microRNA.
